# Targeted Molecular Magnetic Resonance Imaging Detects Brown Adipose Tissue with Ultrasmall Superparamagnetic Iron Oxide

**DOI:** 10.1155/2018/3619548

**Published:** 2018-10-10

**Authors:** Qingqiao Hu, Xiangxun Chen, Juan Liu, Wenjuan Di, Shan Lv, Lijun Tang, Guoxian Ding

**Affiliations:** ^1^Department of Nuclear Medicine, The First Affiliated Hospital of Nanjing Medical University, Nanjing 210029, Jiangsu, China; ^2^Department of Radiology, The First Affiliated Hospital of Nanjing Medical University, Nanjing 210029, Jiangsu, China; ^3^Department of Geratology, The First Affiliated Hospital of Nanjing Medical University, Nanjing 210029, Jiangsu, China

## Abstract

The peptide (CKGGRAKDC-NH2) specifically targets the brown adipose tissue (BAT). Here we applied this peptide coupled with polyethylene glycol (PEG)-coated ultrasmall superparamagnetic iron oxide (USPIO) nanoparticles to detect BAT in vivo by magnetic resonance imaging (MRI). The peptide was conjugated with PEG-coated USPIO nanoparticles to obtain targeted USPIO nanoprobes. Then the nanoprobes for BAT were evaluated in mice. T2⁎-weighted images were performed, precontrast and postcontrast USPIO nanoparticles. Finally, histological analyses proved the specific targeting. The specificity of targeted USPIO nanoprobes was observed in mice. The T2⁎ relaxation time of BAT in the targeted group decreased obviously compared to the controls (P<0.001). Prussian blue staining and transmission electron microscope confirmed the specific presence of iron oxide. This study demonstrated that peptide (CKGGRAKDC-NH2) coupled with PEG-coated USPIO nanoparticles could identify BAT noninvasively in vivo with MRI.

## 1. Introduction

Obesity has become globally epidemic. It is associated with an increased risk for many chronic diseases, such as diabetes, hypertension, and heart disease. Obesity will develop when energy intake exceeds expenditure [1].

As known, white adipose tissue (WAT) is the main site of energy storage in the form of triglycerides, while brown adipose tissue (BAT) is the major depot of adaptive thermogenesis in mammals [2]. BAT, which is mainly located in intrascapular and paraspinal region in rodents and humans [3], plays a key role in the balance of energy metabolism. The increase in the amount or activity of BAT can effectively increase the energy consumption, reduce the WAT accumulation, improve metabolism, and resist obesity and hyperlipidemia as well as other metabolic disorders [4].

The functional activity of BAT has been researched a lot in animals; then extrapolation to humans of results of these studies firstly needs noninvasive approaches to assess human BAT [5]. The positron emission tomography/computed tomography (PET/CT) imaging with 2-deoxy-2-[18F]fluoro-D-glucose ([18F]DG) is the most common technique for BAT imaging [6]. However, the PET-CT imaging depends on high glucose utilization which is associated with metabolic activity. [18F]DG PET-CT imaging only shows activated BAT and is not sensitive to BAT in the nonactivated thermoneutral state. Another limitation of PET imaging is owing to the use of ionizing radiation [7].

Recently, molecular imaging has arisen to visualize and characterize BAT at the molecular as well as cellular level with different imaging modalities that include positron emission tomography, single photon emission computed tomography, and optical imaging. Because of the high spatial resolution and simultaneous anatomic, physiologic, and functional information, magnetic resonance imaging (MRI) is one of the best noninvasive methods in molecular imaging for assessing function of tissues or diagnosing diseases [8].

Various nanoparticulate contrast agents for MR molecular imaging have been developed, like ultrasmall superparamagnetic iron oxide (USPIO), lutetium oxide nanoparticles, and so on [9–11]. With smaller particle, high biocompatibility, and low toxicity, USPIO has become an attractive contrast agent in molecular imaging. USPIOs possess ultrasmall size as well as superparamagnetic properties and can produce large magnetism in the weak external magnetic field. In vivo, USPIO will result in the nonuniformity of local magnetic field which can lead to rapid dephasing of proton. So USPIOs can shorten transverse relaxation time and induce signal decrease on T2*∗*-weighted MR images [12].

Since the peptide (CKGGRAKDC) that targeted WAT vasculature was found by Kolonin MG et al., we researched this peptide for over ten years [13]. Our previous study identified the peptide-drug conjugate (CKGGRAKDC-BVT.2733) protecting against diet-induced obesity, and amino-modified peptide (CKGGRAKDC-NH2) specifically homing to BAT [14]. We have achieved two national patents: a targeted peptide for BAT (Patent No. 201010159916.3); a new targeted drug to BAT for treatment of obesity (Patent No. 201010159909.3).

In this study, we innovatively conjugated the targeted peptide (CKGGRAKDC-NH2) with polyethylene glycol (PEG)-coated USPIO nanoparticles to synthesize BAT-targeted USPIO nanoprobe and investigated its potential application in detecting BAT with MRI in vivo.

## 2. Materials and Methods

### 2.1. Animals

C57BL/6J mice were purchased from Nanjing Biomedical Research Institute of Nanjing University. The weight of mice was 27.3±1.1g. The mice were housed five per cage in a room kept at 23 ± 1°C with 12-h light/dark cycle at Animal Core Facility of Nanjing Medical University and were allowed free access to water and food. All animal designs and studies were approved by the Animal Care and Use Committee of Nanjing Medical University.

### 2.2. Peptide and USPIO Nanoparticles

The targeted peptide CKGGRAKDC-NH2 was synthesized by GL Biochem (Shanghai) Ltd. USPIO nanoparticles were commercially available and purchased from Nanjing Nanoeast Biotech Co.

The morphological characters of USPIOs were examined by transmission electron microscopy ([TEM] JEM-200CX, Japan). The nanoparticle size and distribution were calculated by measuring the diameters of 300 particles at least. The hydrodynamic diameter and Zeta potential were analyzed by dynamic light scattering ([DLS] Zetasizer Nano-z, UK). The magnetic saturation moment was measured by Vibrating Sample Magnetometer (Lakeshore7407, USA). To determine the relaxivity of nanoparticles, USPIO nanoparticles were diluted to the concentration of 0, 0.01395, 0.0279, 0.0558, 0.1116, 0.2232, and 0.4464mmol/L and performed on Siemens Prisma 3.0 T MR scanner (Erlangen, Germany). The detailed image parameters were repetition time (TR) = 10000ms and echo time (TE) = 12, 24, 48, 96, 192, 384ms. T2 relaxivity (r2) was plotted against the iron concentration in the nanoparticles dilutions. T2 relaxation rate (r2) was determined by a linear fit.

### 2.3. Synthesis of Targeted USPIO Nanoprobes

The USPIO nanoparticles were coated by PEG. First, 5mg PEG-USPIO nanoparticles (1mg/mL) were taken; then 1mg 1-ethyl-3-(dimethylaminopropyl) carbodiimide hydrochloride (EDC) molecule and 0.5 mg N-Hydroxysuccinimide (NHS) molecule were added. They reacted at a constant temperature shaker for 25 min (25°C, 180 rpm) to activate PEG-USPIO nanoparticles. 2 mg peptide and 5 mg activated PEG-USPIO nanoparticles were mixed and reacted at a shaker for 2 hours. Finally, the unreacted peptide was isolated by using molecular sieves and targeted USPIO nanoprobes were obtained. PEG-USPIO nanoparticles were untargeted and used as control.

### 2.4. In Vivo MRI Scanning

Studies were performed on a 7T preclinical animal MRI scanner by using a dedicated mouse body coil with a coil diameter of 40 mm (Bruker BioSpin, Germany). The animals were maintained under anesthesia with 2%—3% isoflurane in O2 gas and the flow rate was 0.8L/min. In addition, respiratory rates of the mice were monitored to maintain 30 breaths per minute.

T2*∗*-weighted images were acquired by using the multi-gradient-echo (MGE) sequence with free-breath. Detailed image parameters were as follows: repetition time (TR) = 400 ms, echo time (TE) range = 2.7–37.0 ms, echo spacing = 3.12ms, flip angle = 25°, echoes = 12, matrix = 192×192, field of view (FOV) = 30 × 30mm^2^, slice thickness = 0.5 mm, and number of excitations (NEX) = 4.

The mice were randomly divided into two groups: targeted group received targeted USPIO nanoprobes and control group injected with untargeted USPIO nanoparticles at the same dose of 80 *μ*mol Fe/kg body weight through the mice tail vein. First, we repeated the T2*∗*-weighted images before and at 30min, 60min, 90min, 120min, and 180min after injection (n = 2 per group) to research when maximum negative enhancement effect was observed and disappeared. Then we continued to study the specificity of nanoprobes by imaging before and at 120min after intravenous administration of contrast agents on the basis of the results (n = 7 per group).

### 2.5. Image Analysis

The T2*∗*MR relaxometry maps were generated automatically on 7T MRI. Later, T2*∗* value was measured on T2*∗*MR relaxometry maps with FireVoxel software. Region of interest (ROI) was manually drawn surrounding the BAT and WAT or at the center of muscle and ROI on muscle was chosen at the same slice of BAT. ΔR2*∗* was calculated by equation: ΔR2*∗* = R2*∗*post - R2*∗*pre and R2*∗*[s^−1^] = 1/T2*∗*[milliseconds] × 1000.

### 2.6. HE Staining and Prussian Blue Staining

For hematoxylin and eosin (HE) and Prussian blue staining, tissue was fixed in 10% formalin and embedded into paraffin blocks. Thin sections were cut off and deparaffined and rehydrated. The staining was performed by using a routine protocol. Images were obtained with light microscopy (LeicaDFC450C, Germany).

### 2.7. Transmission Electron Microscopy

For TEM analyses, Tissue samples (less than 1mm^3^) were fixed in 5% glutaraldehyde for 2 hours, then fixed in 1% OsO4 for 2 hours and stained with 2% uranyl acetate, dehydrated with acetone, and embedded in EPON resin. Images of ultrathin sections were analyzed with TEM at 60 kV (Hitachi H-7500, Japan).

### 2.8. Statistical Analysis

The data were recorded as the mean ± standard deviation (SD). Statistical comparisons were performed by a one-way analysis of variance (ANOVA) with SPSS19.0. P values of <0.05 were considered statistically significant.

## 3. Results

### 3.1. Characterization of Untargeted USPIO Nanoparticles and Targeted USPIO Nanoprobes

**Figure 1:**
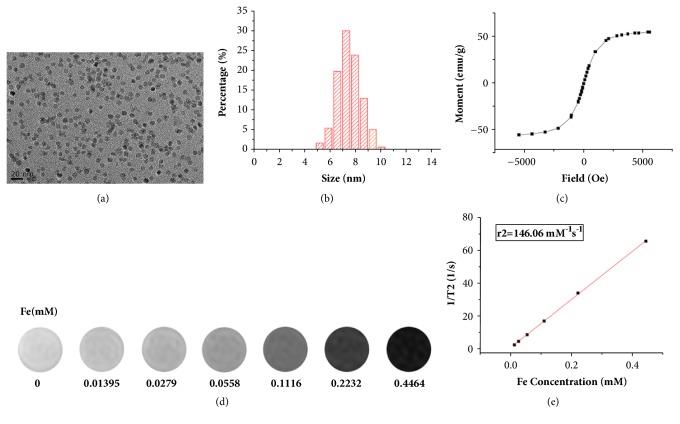
Characterization of the USPIOs. (a) TEM image of the USPIO nanoparticles. (b) Distribution of USPIO core size. (c) Magnetization curve of USPIO nanoparticles. (d) Concentration-dependent T2-mapping of USPIO nanoparticles on 3.0 T MR in vitro. (e) T2 relaxation time at various iron concentrations. Data are recorded as the mean ± SD. TEM indicates transmission electron microscope.

TEM images showed that the USPIOs were well dispersed (Figure 1(a)) and the mean core size was 7.56±0.92 nm (Figure 1(b)). Research suggests that the size-dependent magnetism is important for biomedical application [15]. The saturation magnetization value was 55 emu/g (Figure 1(c)), which was high enough to achieve significant superparamagnetism. With the concentration of USPIO increased, T2-mapping MR signal was gradually attenuated (Figure 1(d)), and the r2 value of USPIOs was 146.06mM^−1^·S^−1^ (Figure 1(e)). The hydrodynamic diameters of untargeted USPIO nanoparticles and targeted USPIO nanoprobes were 17.50±5.90 nm and 20.55±5.70nm, respectively, which were within the range of ultrasmall particles of iron oxide (10-40nm) [16]. Zeta potentials of untargeted USPIO nanoparticles and targeted USPIO nanoprobes were -33.9±6.72 mv and -22.0±5.86 mv, respectively, demonstrating the stability [17].

The targeting specificity of the peptide (CKGGRAKDC-NH2) homing to BAT was proved by confocal immunofluorescence analysis (see Figures S1 in the Supplementary Material).

### 3.2. In Vivo MRI Studies

**Figure 2:**
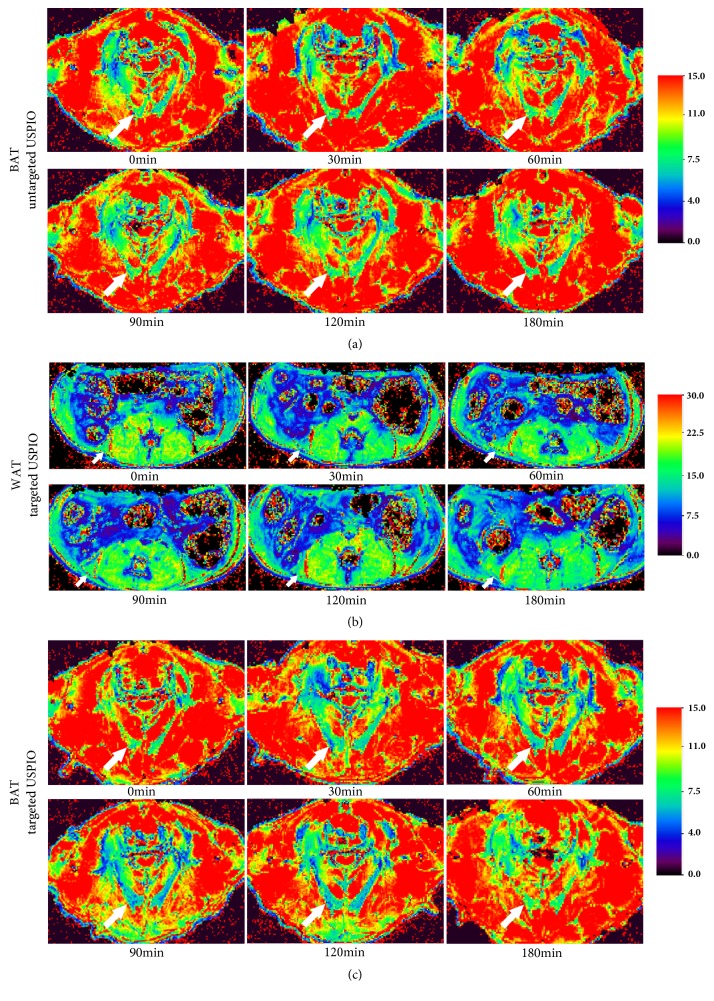
Representative color-coded MR T2*∗* map. T2*∗* map of BAT injected with untargeted USPIO (a). T2*∗* map of WAT (b) and BAT (c) received targeted USPIO (T2*∗*WI indicates T2*∗*-weighted image). Arrows point to BAT in (a) and (c) and point to white adipose tissue in (b).

**Figure 3:**
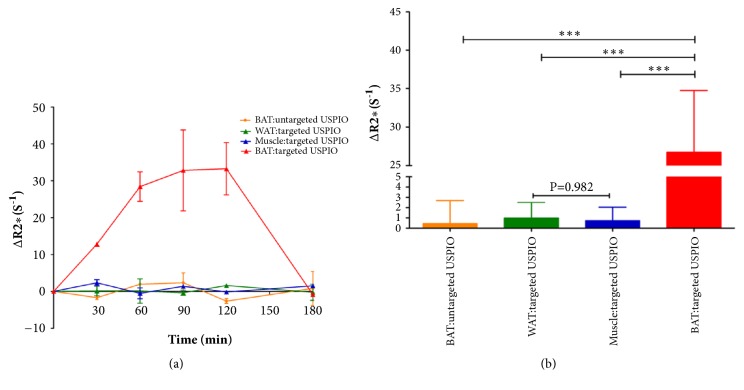
Quantitative analysis of ΔR2*∗* values. (a) The ΔR2*∗* values curve of BAT, WAT, and muscle in targeted group and BAT in control group injected with targeted USPIO or untargeted USPIO, respectively (n = 2). The error bars represent the SD of ΔR2*∗* for two mice in different group. (b) Statistic analysis of ΔR2*∗* values in the BAT in control group and WAT, muscle, and BAT in targeted group (n = 7). *∗∗∗* P<0.001. The error bars represent the SD of ΔR2*∗* for seven mice in different group. Data are recorded as the mean ± SD.

The ability of targeted USPIO nanoprobe to detect BAT in vivo was assessed by MRI.  Figure 2 was the representative color-coded T2*∗* map before and 30min, 60min, 90min, 120min, and 180min after administrating targeted USPIO nanoprobes or untargeted USPIO nanoparticles. The T2*∗* map of BAT in control group (Figure 2(a)) and WAT in targeted group (Figure 2(b)) showed no obvious shortening of the T2*∗* time. BAT in targeted group (Figure 2(c)) exhibited the shortening of T2*∗* time after injection until 120min which is due to the properties of USPIO, and this negative enhancement phenomenon disappeared at 180min. However, no change was observed in muscle. The reduction of T2*∗* time corresponded to the increase in the estimated R2*∗*. The calculated ΔR2*∗* (Figure 3(a)) curves displayed that the ΔR2*∗* of BAT was ascendant after injecting targeted USPIO nanoprobes and kept in steady for some time until 120min and then declined. The ΔR2*∗* of WAT and muscle in targeted group and the ΔR2*∗* of BAT in control group experienced almost no change. Later, we chose to perform T2*∗*MR imaging on mice at 120min after administration. As observed in Figure 3(b), the ΔR2*∗*(26.71s^−1^) of BAT of mice in targeted group was significantly higher (p ≤ 0.05) than all the other negative controls.

### 3.3. Histological Analyses

**Figure 4:**
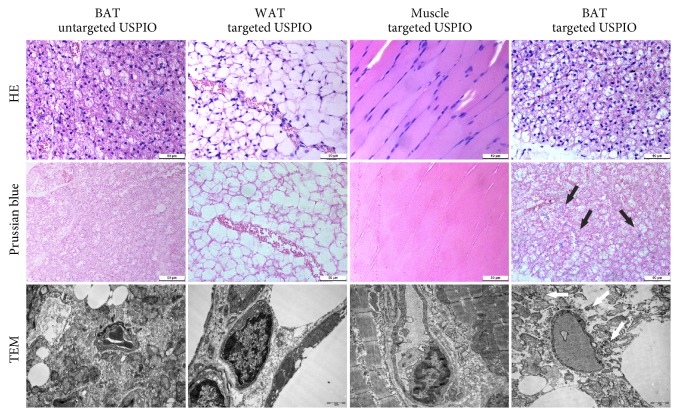
Histological analyses. HE staining, Prussian blue staining, and TEM of BAT in control mice and WAT, muscle, and BAT in targeted group. The arrows refer to the iron particles. Scale bar in staining is 50*μ*m. Scale bar in TEM is 1*μ*m.

Prussian blue staining and TEM analysis have confirmed the MRI results. In Prussian blue staining, iron particles appeared as blue stain and only existed in BAT, not in WAT or muscle in targeted group. As expected, the BAT in control group was Prussian blue negative. Transmission electron microscopy demonstrated that iron particles were located in BAT while no obvious particles were found in the controls, which were consistent with the results of Prussian blue staining and MRI results (Figure 4).

## 4. Discussion

In this study, we innovatively used targeted peptide (CKGGRAKDC-NH2) coupled with USPIO nanoparticles to specifically identify BAT in vivo on MR. According to our knowledge, it has not been researched before. In previous research, a noninvasive method to image BAT with superparamagnetic iron oxide nanoparticle (SPIO) has been used [18]. However, the applicability is limited without BAT-specific probes.

With intrinsic unique magnetic properties that can lead to rapid dephasing of protons, SPIOs offer sufficient sensitivity for T2*∗*-weighted MR imaging. However, their hydrodynamic diameter is >50 nm, resulting in a fast clearance rate by phagocytic cells [19]. Then, USPIO is smaller than SPIO and also has high relaxivity. So USPIO nanoparticles have become an attractive targeted contrast agent for T2*∗*-weighted MR imaging [20]. In our study, the mean hydrodynamic diameter of our USPIOs was about 17.50nm, which helps nanoparticles escape from phagocytes and maintain a higher blood half-life [19]. After measuring, there were about 10^4^ Fe atoms and 230 peptides per nanoparticle. And the calculated amount of targeted ligand per unit area was similar to that of Kazmierczak et al.'s study [21]. The saturation magnetization value (55 emu/g) and the relaxation coefficient r2 of USPIOs (146.06mM^−1^·S^−1^) were high enough to cause obvious T2 contrast enhancement [22, 23]. Due to their strong relaxation properties, low dose of contrast agent (80 *μ*mol Fe/kg body weight) could be detected and was used in our study [24].

USPIO nanoparticles used in this study were stabilized with PEG. The stabilization can prevent aggregation and cleared by macrophages [25]. The Zeta potential of our USPIOs demonstrated that they were stable [17]. PEG is one of the best hydrophilic and biocompatible polymeric coating materials, which can make nanoparticles become water-soluble and does not change the magnetic abilities [26]. At the same time, using PEG to coat USPIO can increase biocompatibility and blood circulation time. In addition, PEG will provide active functional carboxylic acid groups able to conjugate with targeted peptides, which also has been reported by Roberts [27].

Furthermore, recent studies have verified that specific peptide can be linked with USPIO for molecular MR imaging and the peptide properties and functionality as well as the relaxation abilities of USPIO nanoparticles can also be remained [28]. Our research also demonstrated that the targeted peptide (CKGGRAKDC-NH2) conjugated with USPIO can also retain its affinity for BAT.

T2*∗*-weighted imaging was used in our study and acquired with multi-gradient-echo sequence. The signals were acquired at different TE and an appropriate model was fitted to calculate the T2*∗* value, which reflected tissue characterization [29]. USPIO will result in the nonuniformity of local magnetic field. As it happens, T2*∗*WI is sensitive to local field variation and iron oxide nanoparticle detection [30].

It has been confirmed that this MR targeted USPIO nanoprobe could identify BAT in vivo noninvasively and efficiently. The results of our study revealed that targeted USPIO nanoprobe mainly caused T2*∗* signal decrease in BAT of mice on MR relaxometry T2*∗* map. In contrast, there was no T2*∗* signal reduction in BAT injected with untargeted USPIO nanoparticles (Figure 2). The T2*∗* value of WAT was higher than BAT and similar to muscle; in order to observe the difference between WAT and muscle better, the scale range in Figure 2(b) was larger than others. After exploring, we found that when the center value of scale bars was similar to the T2*∗* value of objective tissue, the tissue contrast was the best. In view of Kuhlpeter's report, the R2*∗* value, reciprocal of T2*∗* value, was shown to be proportional to the iron concentration in tissue [31]. Quantitative R2*∗* analysis will enable noninvasive estimation of iron in tissue. Therefore, R2*∗* and ΔR2*∗* value were calculated to perform quantitative analysis in this research. The obvious positive ΔR2*∗* value demonstrated the existence of iron, which was only observed in BAT injected with targeted USPIO nanoprobes. Statistical analyses also proved that the ΔR2*∗* was significantly higher at 120min after administrating targeted USPIO nanoprobes (P<0.05), while the controls showed no substantial deviation from zero. Prussian blue staining and TEM confirmed the specific existence of iron in BAT too.

As shown in Figure 3(a), the maximum negative enhancement effect occurred at 120min and disappeared at 180min postcontrast targeted USPIO nanoprobes. Species of mice, targeted tissue, peptide sequence, and dose of contrast agent were all likely to affect the time when maximum negative enhancement effect occurred and the duration it may continue. Burtea et al. studied USPIO conjugated with targeted peptides to probe pancreatic beta cells [32]. They reported that the lowest signal was attained at 53min and the enhancement still lasted until they stopped to perform MRI at 133min after injection. The dose was similar to ours, but the species of mice, targeted tissue, and sequence of peptide were all different from ours, which might be the cause of different enhancement time. You et al. discovered maximum contrast displayed at 3h and disappeared at 6h after administration of the probe when researching targeted USPIO to detect tumor [33]. In You's study, single-chain oligonucleotide fragment instead of peptide was used. Species of mice and targeted tissue were also different from ours. In addition, the amount of contrast was much higher than ours. These differences might be the reason that the maximum enhancement was generated later and continued longer.

Several limitations may influence the outcome of this study. First, MR T2*∗* relaxometry for quantitative analysis is affected by large-scale field inhomogeneities, which may lead to signal loss as well as overestimation of relaxation rate R2*∗*. Second, the T2*∗* of BAT is relatively short. Theoretically, compared to long T2*∗* values, short T2*∗* values may have more defects. With the shortening of the interecho times, R2*∗* may be highly calculated. If echotimes are very short, the relaxation signal will do not behave exponentially. These defects are based on scanner limitations and the T2*∗* value of tissue [34]. Third, the signal curves were just acquired from two mice in our study, which needed more mice to further verify maximum enhancement time and contrast disappearance time.

The further objective is to detect BAT function on MRI with this probe, which needs more research. Also, whether this probe can recognize BAT in human will be evaluated in future studies.

## 5. Conclusions

In summary, we have demonstrated that MRI with peptide (CKGGRAKDC-NH2) coupled with PEG-coated USPIOs allowed noninvasive assessment of BAT in vivo, which is a specific, sensitive method for imaging BAT and provides new evaluation means for clinical obesity research as well as treatment.

## Data Availability

The research article data used to support the findings of this study are included within the article and the supplementary information file.

## Supplementary Materials

Supplementary Materials In vivo fat homing of the CKGGRAKDC–NH2 motif in C57BL/6J mice. FITC-CKGGRAKDC-NH2 peptide was administrated intravenously in C57BL/6J mice; then different tissues were collected and processed for distribution assay. The green immunofluorescence in formalin-fixed paraffin section is the most obvious in BAT, but nearly not detectable in WAT, liver, or muscle. Scale bar, 50*μ*m.
