# Supplementary figures and images for: Targeted Molecular Magnetic Resonance Imaging Detects Brown Adipose Tissue with Ultrasmall Superparamagnetic Iron Oxide
Source: Biomed Res Int. 2018 Oct 10;2018:3619548. doi: 10.1155/2018/3619548 (PMC6199858; doi:10.1155/2018/3619548)

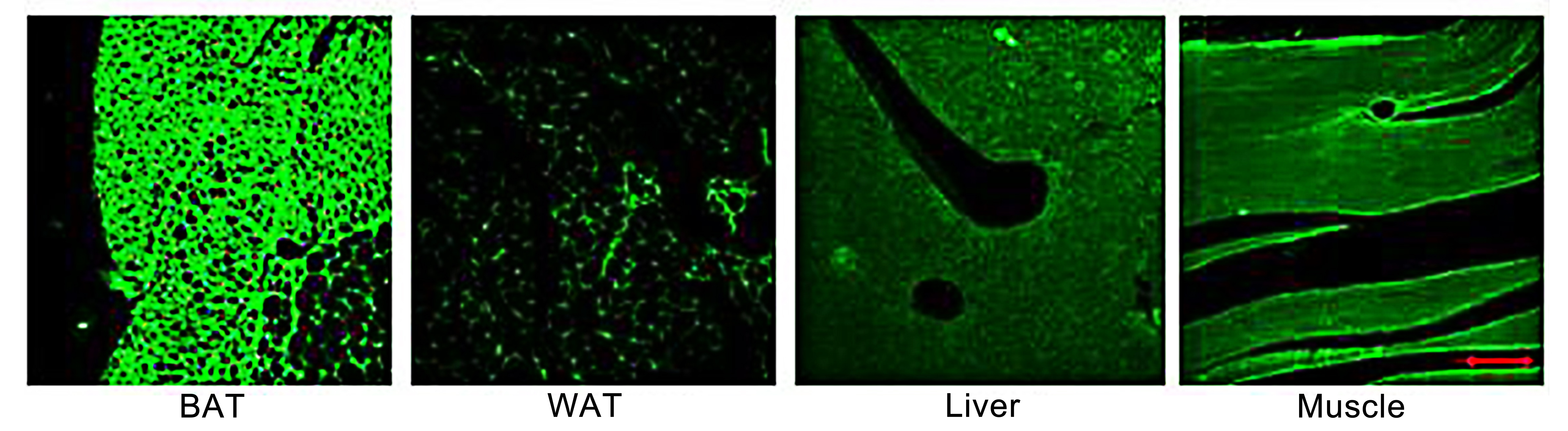

Supplement: Supplementary Materials — In vivo fat homing of the CKGGRAKDC–NH2 motif in C57BL/6J mice. FITC-CKGGRAKDC-NH2 peptide was administrated intravenously in C57BL/6J mice; then different tissues were collected and processed for distribution assay. The green immunofluorescence in formalin-fixed paraffin section is the most obvious in BAT, but nearly not detectable in WAT, liver, or muscle. Scale bar, 50μm. [file 3619548.f1.jpg]
